# Estimating occupancy dynamics for large‐scale monitoring networks: amphibian breeding occupancy across protected areas in the northeast United States

**DOI:** 10.1002/ece3.1679

**Published:** 2015-09-27

**Authors:** David A. W. Miller, Evan H. Campbell Grant

**Affiliations:** ^1^ Department of Ecosystem Science and ManagementPennsylvania State UniversityUniversity ParkPennsylvania16802; ^2^ U.S. Geological SurveyPatuxent Wildlife Research CenterSO Conte Anadromous Fish Laboratory1 Migratory WayTurners FallsMassachusetts01360

**Keywords:** *Ambystoma maculatum*, hierarchical models, *Luthobates sylvaticus*, occupancy, population trend

## Abstract

Regional monitoring strategies frequently employ a nested sampling design where a finite set of study areas from throughout a region are selected and intensive sampling occurs within a subset of sites within the individual study areas. This sampling protocol naturally lends itself to a hierarchical analysis to account for dependence among subsamples. Implementing such an analysis using a classic likelihood framework is computationally challenging when accounting for detection errors in species occurrence models. Bayesian methods offer an alternative approach for fitting models that readily allows for spatial structure to be incorporated. We demonstrate a general approach for estimating occupancy when data come from a nested sampling design. We analyzed data from a regional monitoring program of wood frogs (*Lithobates sylvaticus*) and spotted salamanders (*Ambystoma maculatum*) in vernal pools using static and dynamic occupancy models. We analyzed observations from 2004 to 2013 that were collected within 14 protected areas located throughout the northeast United States. We use the data set to estimate trends in occupancy at both the regional and individual protected area levels. We show that occupancy at the regional level was relatively stable for both species. However, substantial variation occurred among study areas, with some populations declining and some increasing for both species. In addition, When the hierarchical study design is not accounted for, one would conclude stronger support for latitudinal gradient in trends than when using our approach that accounts for the nested design. In contrast to the model that does not account for nesting, the nested model did not include an effect of latitude in the 95% credible interval. These results shed light on the range‐level population status of these pond‐breeding amphibians, and our approach provides a framework that can be used to examine drivers of local and regional occurrence dynamics.

## Introduction

Substantial evidence suggests that amphibian declines are occurring worldwide (Wake 1991; Stuart et al. 2004; Adams et al. 2013). As is the case for many taxa, this assessment has been built on the aggregate of observations from a series of separate local studies of population dynamics and expert assessments used to infer large‐scale population status. Our objective here is to describe a more synthetic alternative for studying large‐scale occurrence dynamics, which allow for data from multiple study areas to be analyzed under a single unified framework. We show how this approach can be used to strengthen conclusions about both regional and local population dynamics.

**Figure 1:**
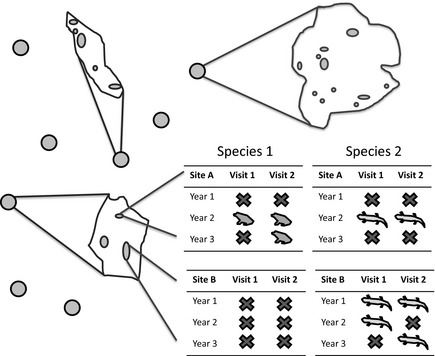
As typical of many large‐scale studies, we collected data using a hierarchical sampling design. We sampled for breeding evidence for two species of amphibians at randomly selected wetlands that occurred in representative sample of protected areas in the northeastern United States. In addition to this hierarchical spatial design, samples also occurred within a temporal nested design, with data collected across 8 years with multiple visits occurring within each year.

Frequently, conservation scientists need to make conclusions about changes in population status across large scales, such as the entire range over which that species occurs. Observations for a single site or for a few closely occurring sites have limited value for making inference outside of the local area at which they are conducted. At the same time, it is often prohibitively costly to randomly sample individuals or points across regional scales. As an alternative, many large‐scale monitoring programs rely on hierarchical sampling designs where a series of smaller areas are selected for study, and sites are randomly chosen and intensively subsampled from within these areas (Fig. 1). Although it is possible to analyze results from each study area separately, stronger inferences are possible if information is aggregated across individual study areas to simultaneously examine both local and regional variation in population dynamics.

Hierarchical study designs are commonly used to address ecological questions. For example, traditional analyses, such as nested ANOVA with subsampling, have a long history of dealing with the hierarchical nature of data collection. More recent developments in mixed effect modeling have increased the utility of analyzing hierarchical data for a range of applications (Bolker et al. 2009). Although now commonplace in many ecological fields, analyses that account for hierarchical sampling designs have been rare for many demographic studies (Schwarz 2002). Examples include methods used for survival analysis, capture–mark–recapture analysis, and occupancy analysis rely on complex likelihoods that do not easily allow for inclusion of random effects or other approaches for apportioning variance components in nested designs (Schwarz 2002; Williams et al. 2002). The computational challenges associated with including random effects in likelihood‐based demographic methods has meant they have lagged behind other analyses in addressing the hierarchical way in which much ecological data are collected.

Recent advances in fitting hierarchical models using Markov chain Monte Carlo (MCMC) have opened the door for analyses of demographic data that account for nested structures (Gelman and Hill 2006; Royle and Dorazio 2008; King et al. 2010; Kéry and Schaub 2012) and allow the inclusion of random effects to apportion variance components in nested sampling designs. Implementation of random effects in demographic analyses has most often focused on accounting for individual‐level variation in parameters (e.g., King et al. 2006; Ford et al. 2012; Miller et al. 2012). Individual heterogeneity is a significant issue in many cases, and these advances have been important in addressing this source of variability (e.g., Cam et al. 2002). In the case of occupancy models, recent attention has also focused on dealing with the nested nature of occurrence, where occurrence at one level is dependent on occurrence at a higher level (e.g., Nichols et al. 2008; McClintock et al. 2010; Mordecai et al. 2011; Miller et al. 2012). Less effort has been devoted to dealing with the variance and covariance that occurs within and among groups when using spatially nested sampling designs. Perhaps the best examples of accounting for random effects in among‐group variation in the population analysis literature are the approaches developed for modeling occupancy of multispecies communities (Dorazio and Royle 2005; Dorazio et al. 2006; Kéry and Royle 2008). In these models, among‐species variation is accounted for using random effects. We demonstrate how that the same approach can and should be used to characterize among‐group variation that emerges from hierarchical spatial sampling.

We analyzed data on the occurrence of breeding wood frog (*Lithobates sylvaticus*) and spotted salamander (*Ambystoma maculatum*) populations from 2004 to 2013 in randomly selected vernal pools that occurred within 14 federally protected lands in the northeastern United States (Fig. 1). Vernal pools (small, isolated temporary wetlands) are important for maintaining biodiversity by providing breeding and foraging habitats and water sources for many wildlife species (Colburn 2004). In particular, vernal pools provide essential breeding and larval habitat for wood frogs and mole salamanders (*Ambystoma* species). Amphibians breeding in vernal pools may be at particular risk of population decline, as the breeding habitat's small size, short hydroperiod, and isolated nature make them more difficult to locate and protect. Both the wood frog and the spotted salamander breed in vernal pools in proximity to upland forest habitat and are widespread in the eastern United States. The southern extent of the wood frog range is found in the southern Appalachian Mountains of Georgia, while the spotted salamander occurs throughout all of the southeast except Florida (Lannoo 2005). Occupancy rates are lower in habitats in urban landscapes than in intact hardwood forests, and occurrence of both species is maximized in short‐duration hydroperiod wetlands (Rubbo and Kiesecker 2005). These are among the first species to breed in the spring, and their vernal pool habitats are typically dry by mid‐summer (Colburn 2004); thus, their breeding activity and success is dependent on precipitation and pool hydroperiod (Brooks 2004).

We fit hierarchical occupancy models (MacKenzie et al. 2006; Kéry and Royle 2008; Royle and Dorazio 2008; Zipkin et al. 2012) using observations of breeding effort for each species. We demonstrate two approaches to the analysis. Our first goal (and the stated goal of many monitoring programs) was to simply estimate the observed trends in occupancy at area and regional levels. Estimating annual changes in occupancy using linear trends also allowed us to explore factors that may explain different occupancy trajectories among areas. Specifically, we hypothesized that a latitudinal gradient in occupancy trends was present. In the northeastern United States, latitude is a good proxy for two factors we hypothesized would have negative effects on occupancy: urbanization and the expected impact of climate change. In both cases, we expected a greater negative effect in the southern portion of the region where human population densities are much higher, and in the southern end of the species' ranges where warming and drying are likely to have a greater influence on habitat occupancy. Our second goal was to fit models using a dynamic occupancy approach, in which information was shared across refuges about the distribution of transition probabilities and annual differences in these probabilities. This allowed us to make inferences about year‐to‐year changes in occupancy at both the study area and regional level.

## Materials and Methods

### Data collection

We conducted repeat‐visit surveys to assess the presence of breeding wood frogs and spotted salamanders in vernal pool wetlands during the spring of 2004–2013. Sites were selected using a nested sampling design with the goal of understanding local and regional dynamics of these species. We collected 2–10 years of data in 14 federally protected areas from throughout the region (Table 1). Protected areas were included in the sampling program based on interest of local resource managers. Although areas were not selected randomly, locations are spatially representative of the region and the variety of federally protected areas in the northeastern United States. Protected areas range from northern Maine south to West Virginia, Maryland, and Washington D.C. Within these protected areas, vernal pool wetlands were chosen for sampling by either (1) random selection from a complete sample of vernal pools (if a comprehensive wetland habitat map had been completed) or (2) sampling random points within the area and mapping clusters of vernal pools (Van Meter et al. 2008). Not all areas participated in all years, dependent on annual priorities of the resource manager and funding availability.

**Table 1:** Summary of areas where sampling occurred

Study area	Number of wetlands	Years sampled	Latitude
Acadia National Park	28	2004–2007, 2009–2013	44.4
Cape Cod National Seashore	30	2004–2009, 2011–2013	41.9
Canaan Valley National Wildlife Refuge and State Park	111	2004–2013	39.1
Delaware Water Gap National Recreation Area	45	2005–2006	41.2
Eastern Massachusetts National Wildlife Refuge	39	2004–2012	42.5
Erie National Wildlife Refuge	19	2004–2007, 2009–2013	41.7
Gettysburg National Historic Park	20	2005–2007, 2012	39.8
Great Swamp National Wildlife Refuge	95	2004–2013	40.7
Iroquois National Wildlife Refuge	20	2005–2007, 2012	43.1
Moosehorn National Wildlife Refuge	43	2004–2007, 2010–2011	45.1
Patuxent Research Refuge	100	2004–2013	39.1
Rachel Carson National Wildlife Refuge	12	2004–2014	43.3
Rock Creek Park	11	2005–2013	39.0
Walkill River National Wildlife Refuge	32	2004–2006, 2010‐2013	41.2

Wetlands were sampled in spring to coincide with timing of breeding for the focal species. Timing of visits varied according to latitude to match differences in typical breeding phenologies across the region. Two to four visits were attempted each year, and during each visit, two observers searched each pool for egg masses of wood frogs (*Lithobates sylvatica*) and spotted salamanders (*Ambystoma maculatum*) by walking the perimeter. Each observer independently recorded whether or not they observed egg masses for each species during the visit. For analyses, we considered a detection to have occurred during a visit if at least one observer recorded the species. We recorded date for all visits to be used as a detection covariate to control for potential confounding due to variation in the timing of egg‐laying and potential predation.

### Statistical analysis

To properly account for the nested design under which the data were collected, we analyzed data with hierarchical models fit using a Bayesian framework. The statistical models we use are extensions of basic static and dynamic occupancy models (MacKenzie et al. 2002, 2003), fit in a Bayesian state‐space framework (Royle and Kéry 2007; Royle and Dorazio 2008), and extended to include among‐area and among‐year random effects for the primary parameters in the model. Analyses were analogous to methods used for community modeling of occupancy (Kéry and Royle 2008; Zipkin et al. 2012), where among‐species random effects were replaced by among‐area effects.

We present two separate analyses, each formulated to address different monitoring objectives. The first example uses a static estimator of occupancy (MacKenzie et al. 2002), where information about the occupancy status in the current year is assumed to be independent of the status in the previous year. In this approach, two primary parameters are of interest: *ψ*_*ij*_ – the probability a site in the *i*th area is occupied in the *j*th year; and *p*_*ijk*_ – the probability of detecting egg masses at a site in the *i*th area in the *j*th year during the *k*th visit given the site is actually occupied. This approach satisfies the desire to evaluate changes in occupancy probabilities, but does not explicitly address the dynamic processes that lead to annual changes in occupancy. The second example is based on a dynamic occupancy estimator (MacKenzie et al. 2003), where occupancy of a site in a given year is allowed to depend on the occupancy status in the previous year. The parameterization we used for the detection was identical for both examples, but a different parameterization was used to describe occurrence patterns. In the dynamic approach, only the initial occupancy (*ψ*_*ij*_) for an area is directly estimated. Occupancy in subsequent years is a function of two transition parameters: *γ*_*ij*_ – the probability a site that was unoccupied in the *i*th area in year *j* was occupied in year *j *+* *1 (colonization); and *φ*_*ij*_ – the probability a site that was occupied in the *i*th area in year *j* was occupied in year *j *+* *1 (persistence).

We use a common structure for both approaches, incorporating both fixed and random effects using a logit‐link function (Bolker et al. 2009). We specified all random effects to be normally distributed on a logit scale. We used the same structure for detection in both examples where detection included an intercept (*α*), a fixed effect for date (*β*), and a random effect for differences among combinations of area and year (*δ*). Values of date were centered to have a mean of 0 for each area so that the effect only accounted for within‐area variation. The probability of detection for the *k*th visit to the *i*th area in the *j*th year is specified as follows:

$$\text{logit}\left(p_{ijk}\right)=\alpha +\beta *{\text{date}}_{ijk}+\delta_{ij}$$

where

*δ*_*ij*_ ~ Normal(0, *σ*_*p*_).

Other parameters are specific to the examples and are described in the following sections. We also needed to specify priors for the parameters. In all cases, we specified intercept terms for fixed effects (*α*) to be distributed Uniform (0, 1) on the real scale, fixed slope parameters (*β*) to be Normal (0, *σ *= 100) on a logit scale, and variance terms for random effects (*σ*_*p*_) to be Gamma (0.1, 0.1).

We fit all models using Markov chain Monte Carlo methods to estimate the posterior distribution under each of our models. MCMC simulations were fit using JAGS v 3.2.0 (Plummer 2003) and run using the statistical package runjags (Denwood 2008) in R v. 2.14.0 (R Core Team 2012). We fit three chains of 20,000 samples after an initial burn in period of 5000 samples for each model. Models were checked for convergence based on trace plots and Gelman–Rubin convergence statistics. We include code for each of the models in the bugs language in the supplementary materials.

#### Model 1 – Occupancy trend analysis

We use an implicit approach where occupancy in each year is not conditional on the occupancy state in the previous year. This approach is useful when we are interested in directly modeling how occupancy changes across time rather than the values for the transition parameters (persistence and colonization) governing these annual changes. Our model in this case needs to describe occupancy for the *i*th area in the *j*th year:

$$\text{logit}\left(\psi_{ij}\right)=\alpha_{i}+\delta_{i}*Y_{j}$$

*δ*_*i*_ ~ Normal(*μ*,* σ*)

The model includes an area‐specific intercept, *α*_*i*_, and describes a linear trend across years, where *Y*_*j*_ is the continuous covariate year and has mean value of 0 across all years for each area. A random effect *δ*_*i*_ describes the trend for the *i*th area. Hyperparameters for the random trend effect are the mean trend for all areas, *μ*, and the standard deviation for the among‐area variation in trends, *σ*.

We included a fixed effect to account for the effect of a predictor variable for the area‐specific trend parameters:

(1) $$\text{logit}\left(\psi_{ij}\right)=\alpha_{i}+\left(\beta *L_{ij}+\delta_{i}\right)*Y_{j}$$

L_*i*_ is the latitude of the *i*th area transformed to have a mean of 0 for all areas and *β* is the effect of latitude on trend. We used a similar set of prior probabilities to those used in the first model: logit(*α*) ~ Uniform(0, 1), *β* ~ Normal(0, 100), *μ* ~ Normal(0, 100), and *σ* ~ Gamma(0.1, 0.1).

We examined how including the random effect structure affected the mean and credible intervals of the parameter estimates and the implications of ignoring nonindependence of wetlands within areas. To do this, we ran an additional model without *δ*_*i*_ and compared trend estimates and credible intervals to the model with this term.

#### Model 2 – Annual changes in occupancy

We were also interested in making inferences regarding the dynamic parameters governing annual changes in occupancy. To do this, we used a model where the probability of site occupancy is conditional on the occupancy status in the previous year. The persistence probability (*φ*) is the probability that a previously occupied site in time *t−1* stays occupied (i.e., the species does not go locally extinct) in *t* and the colonization probability (*γ*) is the probability an unoccupied site in time *t−1* becomes occupied in *t*. These transition probabilities were allowed to vary by year and among areas as random effects. This formulation allows functional flexibility in annual changes while taking advantage of information provided by the previous occupancy status of sites. Rather than separately estimate these values for each study area using a fixed effects design, we assumed these probabilities came from a common distribution with a mean value for all areas and years (logit[*α*]) and used hyperparameters to describe variation among areas and years (*δ*_*i*_ and *θ*_*j*_, respectively). Persistence for the *i*th area in the *j*th year *φ*_*ij*_ is equal to

$$\text{logit}\left(\varphi_{ij}\right)=\alpha +\delta_{i}+\theta_{j}$$

where

*δ*_*i*_ ~ Normal(0, *σ*_*δ*_) and

*θ*_*j*_ ~ Normal(0, *σ*_*θ*_).

Colonization was formulated the same way. Noninformative priors were chosen for both so that logit(*α*) ~ Uniform(0, 1) and *σ*_*δ*_ ~ Gamma(0.1, 0.1) and *σ*_*θ*_ ~ Gamma(0.1, 0.1). In addition, we specified the starting occupancy probability *ψ*_*i1*_ in the initial year to vary independently among areas (i.e., no hyperparameters) with a prior specified to be *ψ*_*ij*_ ~ Uniform(0, 1).

This approach allowed us to simultaneously make inferences at both the regional and area levels. We were able to estimate a mean occupancy within the region for each of the years, conditional on the assumption that our sample of areas was representative of the public lands in the region as a whole. The random effects structure treats the wetlands within areas as repeated measures of occupancy status within areas and properly propogates sampling error for areas into regional estimates.

At the same time, simultaneously estimating occupancy for all areas can improve estimates for individual areas. To the extent that shared factors affect dynamics for all areas, this will be picked up by the random effect structure used in models. This provides added information when estimating effects for individual areas. We used k‐fold cross‐validation to test whether this was actually the case (Hooten and Hobbs 2015). We compared predictions from the full hierarchical model to a model where hyperparameters were no longer included and, instead, all parameters were treated as fixed effects and estimated separately for each study area. We used 5‐fold validation and deviance of the predicted observations as our measure of prediction accuracy. This approach allowed us to determine whether our approach truly improved predictions for data that were held out from the primary data set.

## Results

### Model 1

**Figure 2:**
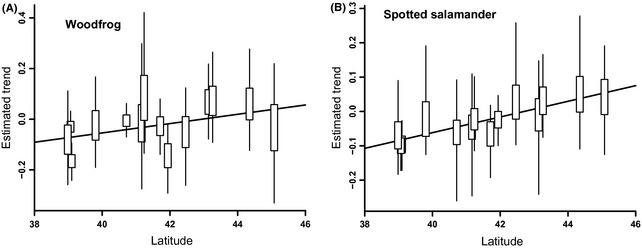
The 50% (box) and 95% (line) credible intervals for the estimated trend effect of wood frog and spotted salamander occupancy for each of the areas using Model 1. Trend effects are the change in occupancy per year on a logit scale. The red line represents the mean of the posterior distribution for the relationship between trend and latitude (β*L_*ij*_ + *δ*_*i*_ from eq. (1) in the text). For both species, we found that on average southern areas were declining more quickly. Trend estimates for individual areas were generally imprecise except for those with the greatest sampling effort.

**Figure 3:**
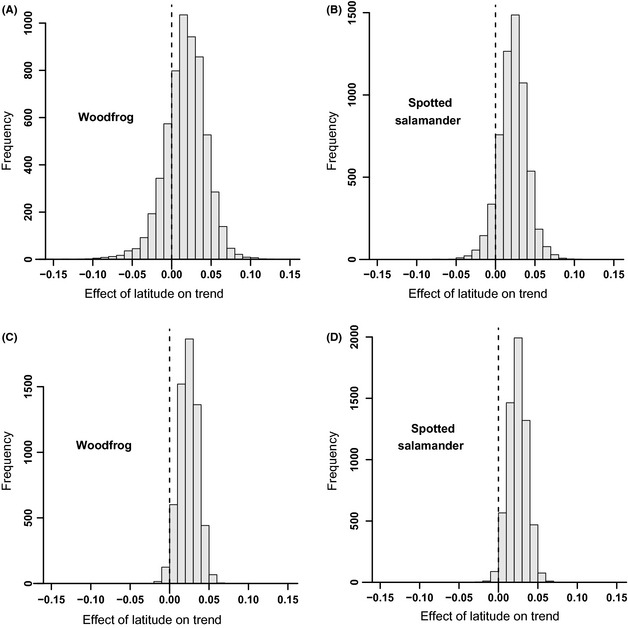
We examined how including the random effect structure influenced posterior distributions for the effect of a one‐degree increase in latitude on trend (*β* from Model 1 in the text). For both species, we found that when the random effect was included for among‐area variation in trend (A and B), the posterior distribution was wider than when the random effect was not included (C and D). Not properly accounting for the nested structure led to overestimation of confidence in estimates.

Estimated trends for individual areas were imprecise in most cases (Fig. 2). Higher precision occurred for areas with a larger number of sites sampled and where sampling occurred annually across the whole study period. Despite this, we did find some support for a latitudinal gradient in trends across all areas (Fig. 3). The 95% credible interval for the estimated effect in each species did include 0 (−0.056 to 0.129 for wood frog and −0.030 to 0.123 for spotted salamanders). The posterior probability of a positive effect of latitude on trend (i.e., consistent with a positive latitudinal gradient in trends; Fig. 3) was 0.835 and 0.916 for wood frogs and spotted salamanders, respectively. While supportive of our hypothesis, the results are not conclusive because of significant overlap of the credible intervals with 0. However, we would have concluded that there was overwhelming support for the relationship if we had not included the random effect in the model and thus treated each wetland as independent despite the nested structure of data collection. Based on narrower credible intervals, we would have estimated a 0.994 and 0.992 probability of a positive relationship (Fig. 3).

### Model 2

**Figure 4:**
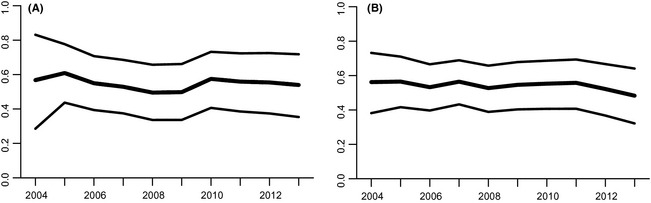
Mean annual regional estimates of occupancy of vernal pool habitats in northeast areas monitored for wood frogs (A) and spotted salamanders (B). Estimates were generated using Model 2.

At the regional level, there appeared to be a slight decline in wood frog occupancy during the middle years of the study period, with the lowest occupancy occurring in 2008 and 2009 (Fig. 4). Mean occupancy for spotted salamanders was stable with perhaps a minor decline in the final year of the study. Interestingly, there does not seem to be a very strong correlation in annual changes between the two species at the regional level, indicating they may respond differently to annual environmental variation or to different drivers.

Overall turnover was higher for the shorter‐lived wood frog than for the spotted salamander. Mean among‐area and among‐year colonization and persistence probabilities for wood frogs were 0.119 and 0.896, respectively, and the mean values for spotted salamanders were 0.085 and 0.933, respectively.

**Figure 5:**
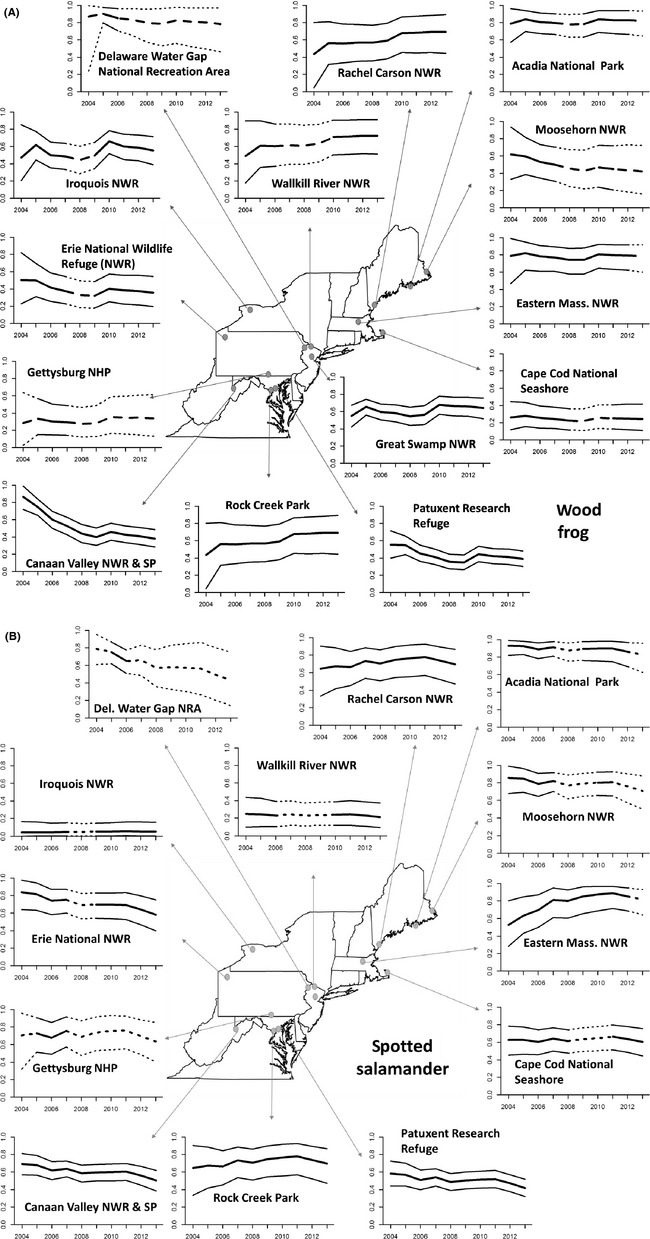
We used hierarchical dynamic occupancy models to estimate annual occupancy probabilities and 95% credible intervals for wood frogs (A) and spotted salamanders (B) for each of the participating areas. We only display data for years data were collected at an area using solid lines and for years when data were not collected using dashed line. Estimates are both influenced by the data collected at the specific study area and by the mean value for all areas because of the random effect structure. Spotted salamanders were never observed at Great Swamp National Wildlife Refuge, and therefore, not estimates are displayed for the species at this site. Estimates were generated using Model 2.

Although regional estimates of occupancy were fairly consistent, there was significant annual variation in occupancy within the individual protected areas (Fig. 5). In some cases, distinct declines occurred, while in others, occupancy increased across the study period. Areas also varied in their mean occupancy, ranging from having low occupancy in all years to those where occupancy was nearer to 100% in all years. Estimates for each of the areas also show characteristics of the overall regional pattern, reflecting the fact that information was shared across areas due to the random effect structure in the analysis. The strongest shrinkage to the regional mean occurred for areas where sample sizes were small. It is possible to generate estimates in years where sampling did not occur for an area. The characteristics of the area during the sampled years and information provided by patterns in other areas provide a basis for making inferences during these years. We distinguish these estimates in Figure 5 using dotted lines. In general, credible intervals are wider in unsampled years where loss of precision increases with the number of years elapsed since the area was last sampled. Interestingly, there are minimal effects on credible intervals for effects on occupancy in areas where only a year or two are missing in between years where an area was sampled.

We found strong support that the hierarchical structure (random effects model) improved predictions when compared to a model where parameters were estimated independently for each study area (fixed effects model). In the case of wood frogs, the deviance for predictions from the hold‐out data sets (5‐fold cross‐validation) was 7213.86 for the random effects model compared to 7255.76 for the fixed effects model (lower deviance indicates better model fit). The improvement was even greater for the spotted salamander model where deviance was 6919.71 for the random effects model compared to 7309.10 for the fixed effects model.

## Discussion

As is the case with many species, despite decades of research on both the spotted salamander and the wood frog, there is little information on their status at regional scales. Instead, our knowledge of their status has had to be extrapolated from smaller‐scale studies. Existing local‐level information suggests that these species, especially wood frogs, exhibit cyclical dynamics in breeding effort. Petranka et al. (2007) found a decline in occupancy of wood frog and less pronounced decline in spotted salamander egg masses from 1996 to 2006 at both restoration and reference sites in a single mitigation site in North Carolina. Using calling survey data, Gibbs et al. (2005) found populations of wood frogs in NY were stable between surveys conducted from 1973 to 1980 and 2001 to 2002. In Wisconsin, Trenham et al. (2003) report stable populations of wood frogs over the period from 1981 to 1998. Occupancy of wetlands in the Chesapeake and Ohio Canal National Historic Park have been relatively stable for both species from 2004 to 2013, although other species have been declining (Grant et al. 2013).

Our expectation was that populations of wood frogs and spotted salamanders occurring in protected areas would exhibit stable occupancy patterns (i.e., no trend in occupancy). Important to this expectation is that, as a metric for population change, we expect occupancy to be less sensitive to annual variability in breeding effort (or cyclic patterns in abundance) than annual abundance. While previous research finds variation in annual abundance, most do not find absence of breeding effort (Berven 1990; Pechmann et al. 1991). Our analyses suggest that on average at the regional level, occupancy probabilities were relatively stable from 2004 to 2013.

However, we did find support for among‐area differences in the occupancy dynamics across the period of study (Figs. 2, 5). Some areas exhibited substantial increases and decreases for both species. Similarly, trends were directionally consistent with our prediction of a north–south gradient in population trends across the region, with greater decreases in the southern end of the sampling region (Fig. 2). The estimated latitudinal gradient in trends (*β*) was greater for wood frogs, which is not surprising given that our study area encompasses the southernmost portion of their range (Lannoo 2005).

Schwarz (2002) noted more than a decade ago that proper accounting for variance components is a significant and unmet challenge for many demographic analyses. Although there are still significant gaps, the development of Bayesian hierarchical and MCMC methods has opened the door to addressing this deficiency. Other examples of recent extensions in the demographic literature include methods to deal with individual variation (Ford et al. 2012); group effects (Zipkin et al. 2009); path analysis (Cubaynes et al. 2012; Gimenez et al. 2012); experimental design (Schwarz 2002); and spatial autocorrelation (Chelgren et al. 2011; Johnson et al. 2013). The strength of many of the demographic analysis methods has always been the ability to properly weight observed samples to account for nondetected (unsampled) individuals. This has often precluded the use of analytical frameworks in many other fields. The great potential of Bayesian methods for demographic analyses is the ability to address these limitations. Our methods make further progress in this area and are likely to be useful for a range of species and monitoring programs that rely on hierarchical sampling efforts.

By designing a monitoring program with multiple protected areas across the northeastern USA, and incorporating this design explicitly in our analysis, we were able to predict occupancy dynamics and characterize regional patterns in trends than by evaluating populations solely at a regional or local level. Hierarchical models offer a powerful approach for analyzing occurrence data collected using nested designs, both properly accounting for the sampling structure and allowing for inference at and across multiple scales. Further, the utility of our nested monitoring design has clear implications for resource management. Concern is often triggered by population status at large scales, while management generally seeks to improve conditions at the local level (Grant et al. 2013). Understanding conditions at the local scale and how they relate to regional patterns is important not only in determining where action is most needed but also to tailor efforts to address local variation. A key step for doing this in our case will be to extend inference to better understand the factors that lead to within‐site and among‐year variation in occupancy dynamics.

## Conflict of Interest

None declared.

## Supporting information


**Data S1.** JAGS code ‐ Model 1.

**Data S2.** JAGS code ‐ Model 2.

## References

[ece31679-bib-0001] Adams, M. J. , D. A. W. Miller , E. Muths , P. S. Corn , E. H. C. Grant , L. L. Bailey , et al. 2013 Trends in Amphibian Occupancy in the United States (ed HYH Chen). PLoS One 8:e64347. 23717602 10.1371/journal.pone.0064347 PMC3661441

[ece31679-bib-0003] Berven, K. 1990 Factors affecting population fluctuations in larval and adult stages of the wood frog (Rana sylvatica). Ecology 71:1599–1608.

[ece31679-bib-0004] Bolker, B. M. , M. E. Brooks , C. J. Clark , S. W. Geange , J. R. Poulsen , M. H. H. Stevens , et al. 2009 Generalized linear mixed models: a practical guide for ecology and evolution. Trends Ecol. Evol. 24:127–135. 19185386 10.1016/j.tree.2008.10.008

[ece31679-bib-0005] Brooks, R. T. 2004 Weather‐related effects on woodland vernal pool hydrology and hydroperiod. Wetlands 24:104–114.

[ece31679-bib-0006] Cam, E. , W. A. Link , E. G. Cooch , J.‐Y. Monnat , and E. Danchin . 2002 Individual covariation in life‐history traits: seeing the trees despite the forest. Am. Nat. 159:96–105. 18707403 10.1086/324126

[ece31679-bib-0007] Chelgren, N. D. , M. J. Adams , L. L. Bailey , and R. B. Bury . 2011 Using multilevel spatial models to understand salamander site occupancy patterns after wildfire. Ecology 92:408–421. 21618920 10.1890/10-0322.1

[ece31679-bib-0008] Colburn, E. A. 2004 Vernal pools: natural history and conservation. McDonald & Woodward Pub Co., Blacksburg, Virginia.

[ece31679-bib-0009] Cubaynes, S. , C. Doutrelant , A. Grégoire , P. Perret , B. Faivre , and O. Gimenez . 2012 Testing hypotheses in evolutionary ecology with imperfect detection: capture‐recapture structural equation modeling. Ecology 93:248–255. 22624306 10.1890/11-0258.1

[ece31679-bib-0010] Denwood, M. J. 2008 Runjags: run Bayesian MCMC models in the BUGS syntax from within R. R package version 9.

[ece31679-bib-0011] Dorazio, R. M. , and J. A. Royle . 2005 Estimating Size and Composition of Biological Communities by Modeling the Occurrence of Species. J. Am. Stat. Assoc. 100:389–398.

[ece31679-bib-0012] Dorazio, R. M. , J. A. Royle , B. Söderström , and A. Glimskär . 2006 Estimating species richness and accumulation by modeling species occurrence and detectability. Ecology 87:842–854. 16676528 10.1890/0012-9658(2006)87[842:esraab]2.0.co;2

[ece31679-bib-0013] Ford, J. H. , M. V. Bravington , and J. Robbins . 2012 Incorporating individual variability into mark‐recapture models. Methods Ecol. Evol. 3:1047–1054.

[ece31679-bib-0014] Gelman, A. , and J. Hill . 2006 Data analysis using regression and multilevel/hierarchical models. Cambridge Univ. Press, Cambridge, Massachusetts.

[ece31679-bib-0015] Gibbs, J. , K. Whiteleather , and F. Schueler . 2005 Changes in frog and toad populations over 30 years in New York State. Ecol. Appl. 15:1148–1157.

[ece31679-bib-0016] Gimenez, O. , T. Anker‐Nilssen , and V. Grosbois . 2012 Exploring causal pathways in demographic parameter variation: path analysis of mark‐recapture data. Methods Ecol. Evol. 3:427–432.

[ece31679-bib-0017] Grant, E. H. C. , E. F. Zipkin , J. D. Nichols , and J. P. Campbell . 2013 A strategy for monitoring and managing declines in an amphibian community. Conserv. Biol., 27:1245–1253. 24001175 10.1111/cobi.12137

[ece31679-bib-0018] Hooten, M. B. , and N. T. Hobbs . 2015 A guide to Bayesian model selection for ecologists. Ecol. Monogr., 85:3–28. 140521113529001.

[ece31679-bib-0019] Johnson, D. S. , P. B. Conn , M. B. Hooten , J. C. Ray , and B. A. Pond . 2013 Spatial occupancy models for large data sets. Ecology 94:801–808.

[ece31679-bib-0020] Kéry, M. , and J. Royle . 2008 Hierarchical Bayes estimation of species richness and occupancy in spatially replicated surveys. J. Appl. Ecol. 45:589–598.

[ece31679-bib-0021] Kéry, M. , and M. Schaub . 2012 Bayesian population analysis using WinBUGS: a hierarchical perspective. Academic Press, San Diego.

[ece31679-bib-0022] King, R. , S. P. Brooks , B. J. T. Morgan , and T. Coulson . 2006 Factors influencing soay sheep survival: a Bayesian analysis. Biometrics 62:211–220. 16542248 10.1111/j.1541-0420.2005.00404.x

[ece31679-bib-0023] King, R. , B. J. T. Morgan , O. Gimenez , and S. P. Brooks . 2010 Bayesian analysis for population ecology. CRC Press, Boca Raton.

[ece31679-bib-0024] Lannoo, M. J. 2005 Amphibian declines: the conservation status of United States species. University of California Press, Berkeley.

[ece31679-bib-0025] MacKenzie, D. , J. Nichols , G. Lachman , S. Droege , J. A. Royle , and C. Langtimm . 2002 Estimating site occupancy rates when detection probabilities are less than one. Ecology 83:2248–2255.

[ece31679-bib-0026] MacKenzie, D. , J. Nichols , and J. Hines . 2003 Estimating site occupancy, colonization, and local extinction when a species is detected imperfectly. Ecology 84:2200–2207.

[ece31679-bib-0027] MacKenzie, D. I. , J. D. Nichols , J. A. Royle , K. H. Pollock , L. L. Bailey , and J. E. Hines . 2006 Occupancy estimation and modeling: inferring patterns and dynamics of species occurrence. Academic Press, New York.

[ece31679-bib-0029] McClintock, B. T. , J. D. Nichols , L. L. Bailey , D. I. MacKenzie , W. L. Kendall , and A. B. Franklin . 2010 Seeking a second opinion: uncertainty in disease ecology. Ecol. Lett. 13:659–674. 20426794 10.1111/j.1461-0248.2010.01472.x

[ece31679-bib-0030] Miller, D. A. W. , B. L. Talley , K. R. Lips , and E. H. Campbell Grant . 2012 Estimating patterns and drivers of infection prevalence and intensity when detection is imperfect and sampling error occurs. Methods Ecol. Evol. 3:850–859.

[ece31679-bib-0031] Mordecai, R. S. , B. J. Mattsson , C. J. Tzilkowski , and R. J. Cooper . 2011 Addressing challenges when studying mobile or episodic species: hierarchical Bayes estimation of occupancy and use. J. Appl. Ecol. 48:56–66.

[ece31679-bib-0033] Nichols, J. , L. Bailey , A. F. O'Connell , N. W. Talancy , E. H. C. Grant , A. T. Gilbert , et al. 2008 Multi‐scale occupancy estimation and modelling using multiple detection methods. J. Appl. Ecol. 45:1321–1329.

[ece31679-bib-0034] Pechmann, J. H. K. , D. E. Scott , R. D. Semlitsch , J. Cadwell , L. Vitt , and J. Gibbons . 1991 Declining amphibian populations: the problem of separating human impacts from natural fluctuations. Science 253:892–895. 17751826 10.1126/science.253.5022.892

[ece31679-bib-0035] Petranka, J. W. , E. M. Harp , C. T. Holbrook , and J. A. Hamel . 2007 Long‐term persistence of amphibian populations in a restored wetland complex. Biol. Conserv. 138:371–380.

[ece31679-bib-0101] Plummer, M. 2003 JAGS: a program for analysis of Bayesian graphical models using Gibbs sampling in Hornik K., Leisch F. and Zeileis A., eds. Proceedings of the 3rd International Workshop on Distributed Statistical Computing. Vienna, Austria.

[ece31679-bib-0037] R Core Team . 2012 R: A language and environment for statistical computing. R Foundation for Statistical Computing, Vienna, Austria.

[ece31679-bib-0041] Royle, J. A. , and R. M. Dorazio . 2008 Hierarchical modeling and inference in ecology: the analysis of data from populations, metapopulations and communities. Academic Press, New York.

[ece31679-bib-0042] Royle, J. A. , and M. Kéry . 2007 A Bayesian state‐space formulation of dynamic occupancy models. Ecology 88:1813–1823. 17645027 10.1890/06-0669.1

[ece31679-bib-0043] Rubbo, M. J. , and J. M. Kiesecker . 2005 Amphibian Breeding Distribution in an Urbanized Landscape. Conserv. Biol. 19:504–511.

[ece31679-bib-0044] Schwarz, C. 2002 Real and quasi‐experiments in capture‐recapture studies. J. Appl. Stat. 29:459–473.

[ece31679-bib-0045] Stuart, S. , J. Chanson , N. Cox , B. Young , A. Rodrigues , D. Fischman , et al. 2004 Status and Trends of Amphibian Declines and Extinctions Worldwide. Science 306:1783–1786. 15486254 10.1126/science.1103538

[ece31679-bib-0046] Trenham, P. , W. Koenig , M. Mossman , S. Stark , and L. Jagger . 2003 Regional dynamics of wetland‐breeding frogs and toads: turnover and synchrony. Ecol. Appl. 13:1522–1532.

[ece31679-bib-0047] Van Meter, R. , L. L. Bailey , E. H. C. Grant , B. County , M. Estuarine , and E. Sciences . 2008 Methods for estimating the amount of vernal pool habitat in the northeastern United States. Wetlands 28:585–593.

[ece31679-bib-0049] Wake, D. 1991 Declining amphibian populations. Science 253:860. 17751819 10.1126/science.253.5022.860

[ece31679-bib-0050] Williams, B. K. , J. D. Nichols , and M. J. Conroy . 2002 Analysis and management of animal populations: modeling, estimation, and decision making. Academic Press, New York.

[ece31679-bib-0051] Zipkin, E. F. , A. DeWan , and J. Andrew Royle . 2009 Impacts of forest fragmentation on species richness: a hierarchical approach to community modelling. J. Appl. Ecol. 46:815–822.

[ece31679-bib-0052] Zipkin, E. F. , E. H. C. Grant , and W. F. Fagan . 2012 Evaluating the predictive abilities of community occupancy models using AUC while accounting for imperfect detection. Ecol. Appl. 22:1962–1972. 23210312 10.1890/11-1936.1

